# Relational values resonate broadly and differently than intrinsic or instrumental values, or the New Ecological Paradigm

**DOI:** 10.1371/journal.pone.0183962

**Published:** 2017-08-30

**Authors:** Sarah C. Klain, Paige Olmsted, Kai M. A. Chan, Terre Satterfield

**Affiliations:** 1 College of Earth, Ocean and Atmospheric Science, Oregon State University, Corvallis, Oregon, United States of America; 2 Institute for Resources, Environment and Sustainability, University of British Columbia, Vancouver, British Columbia, Canada; University of Vermont, UNITED STATES

## Abstract

Value orientations used to explain or justify conservation have been rooted in arguments about how much and in what context to emphasize the intrinsic versus instrumental value of nature. Equally prominent are characterizations of beliefs known as the New Ecological Paradigm (NEP), often used to help explain pro-environmental behaviour. A recent alternative to these positions has been identified as ‘relational value’—broadly, values linking people and ecosystems via tangible and intangible relationships to nature as well as the principles, virtues and notions of a good life that may accompany these. This paper examines whether relational values are distinct from other value orientation and have potential to alleviate the intrinsic-instrumental debate. To test this possibility, we sought to operationalize the construct—relational values—by developing six relational statements. We ask: 1) Do the individual statements used to characterize relational values demonstrate internal coherence as either a single or multi-dimensional construct? 2) Do relational value statements (including those strongly stated) resonate with diverse populations? 3) Do people respond to relational value statements in a consistently different way than NEP scale statements? Data for this work is drawn from an online panel of residents of northeastern US (*n* = 400), as well as a sample of Costa Rican farmers (*n* = 253) and tourists in Costa Rica (*n* = 260). Results indicate relational values are distinct as a construct when compared to the NEP.

## 1. Introduction

Conservation scientists and practitioners have often drawn on ethical constructs to articulate support for policies to protect biodiversity. To those outside the conservation community, it may come as a surprise that the value debate “Why conserve nature?” has become increasingly heated and arguably detrimental despite calls for “a unified and diverse conservation ethic” [1,2]. “Traditional conservationists” advocate for the intrinsic value of nature, protecting nature for its own sake. They often focus on strategies to minimize human interference with ecological processes and invoke ethical and moral arguments to support their stance while being skeptical of corporate involvement in conservation [3]. Such advocates are often pitted against the “new conservationists,” who champion the instrumental value of nature, justifying and prioritizing conservation action based on nature’s benefits to people [4]. New conservationists embrace market-based incentives and collaborating with corporations to protect and enhance the benefits of nature to people (ecosystem services), often derived from human-dominated landscapes [4,5].

Underpinning the intrinsic vs. instrumental debate is a common objective—to promote and encourage conservation actions, from the level of the individual to national governments and international decisions. Marvier [6] and other new conservationists claim that utilitarian conservation arguments do not undermine conservation justifications based on nature’s intrinsic value or an ethical duty to protect biodiversity. Rather, many contend that instrumental arguments offer additional ethical justifications and so “potentially broaden the tent of conservation” [6]. While there is evidence that people hold both instrumental and intrinsic values [7,8], there is reason to believe that appeals to only those types of values can be constraining or possibly alienating to many who may potentially care more and take additional action if environmental issues were framed differently [9,10]. Reducing the importance of nature to only intrinsic or instrumental and monetized value is also not reflective of the largely intuitive ways that people make decisions, understand the world and decide what is right [11–13].

The burgeoning field of ecosystem services (ES)[14], long associated with a purely instrumental perspective, has recently been broadened to include other perspectives on value. The ES concept became globally recognized with the Millennium Ecosystem Assessment [15], which emphasized diverse connections between human well-being and nature, but the category of cultural ES never fit well in the publications that ensued in the following decade [16,17]. The instrumental orientation of ecosystem services is a potential cause of the poor fit, in part because instrumental values are by definition substitutable, whereas cultural values are often not [18,19]. Quantified and/or monetized ES data often omit the more intangible values that “really get at well-being,” [Hannah in 19], such as connectedness and belonging to a community (both human and non-human), sense of place and other culturally and psychologically mediated relationships between people and ecosystems [20]. Consequently, researchers from a wide range of backgrounds, including anthropology, political science, economics, and ecology, have begun to develop methods designed to enable social, cultural and intangible values to play a more prominent role in ES assessments and decision-making without compromising their distinct nature [16,17,19,21–24]. As a result of these and related efforts, the ES field is evolving to the point that the IPBES (Intergovernmental Science-Policy Platform on Biodiversity and Ecosystem Services) conceptual framework has included relational values, which are an additional conception of values, to its mandate [25].

The hope, as argued by Chan et al. [9] is that a relational-value framing will be more inclusive and responsive to known aspects of sources of well-being (e.g., connection to others, place attachment) than instrumental and intrinsic values, particularly when addressing how people make decisions and what they care about. In this case, we refer to framing as in the *framing effect*–deliberate construction of a value statement that may influence the response. The relational “framing” is intended to present value statements such that they facilitate the connection between humans and the natural world.

Relational values encompass “eudaimonic” values—values associated with living a good life as well as reflection about how preferences and societal choices relate to notions of justice, reciprocity, care and virtue [26–29]. They are derived from interactions with and responsibilities to humans, non-humans, landscapes and ecosystems [9]. However, despite these conceptual advances, empirical investigation has been lacking.

Here we test a set of statements aimed to capture and quantify a possible range of social-ecological relations, as a first step toward addressing an overly dichotomized characterization of nature as instrumental or intrinsic. Our hope is the provision of a framework in which instrumental, intrinsic, and other value orientations are not subdivided and can thus be complementary. We pilot several types of social-ecological value statements, including instrumental, intrinsic, and relational value statements as well as value statements that use metaphors to convey a value. We assess whether or not our set of relational value statements demonstrate internal coherence as a single or multi-dimensional construct. We compare responses to relational value statements with additional statements phrased to represent instrumental, intrinsic and metaphorically phrased value statements.

We also address a fundamental question: How do relational values compare to other scales often used to assess strength of environmental commitment? The New Ecological Paradigm Scale (NEP)[30], is the most widely used method to measure beliefs about nature. The NEP aggregates responses to 15 (or as few as 5) statements to assess these beliefes, many of which also characterize people as possessing ecocentric as opposed to anthropocentric beliefs. Social scientists have used the NEP scale with diverse populations and responses have demonstrated variation along the ecocentric-anthropocentric continuum [31].

Although global values surveys using the NEP show variation, research suggests that most people are concerned about the natural world and prefer the notion of “co-existing” with nature rather than dominating it [31]. The NEP largely aligns with an ecocentric vs. anthropocentric framing, by assessing the extent to which people recognize 1) ecological limitations to growth; 2) the importance of maintaining a balance of nature; and 3) rejection of the idea that nature “exists primarily for human use” [32]. Thus, the question remains: does the addition of relational value items add something to the study of environmental beliefs or values, perhaps complementing the NEP by offering a different framing?

This discussion of both value types and their applicability can be summarized as three research questions underpinning our survey design and stated below:

1. Do various types of relational value statements correlate as a single construct?
2. Do relational value statements (including those strongly stated) resonate with (i.e., elicit agreement) amongst diverse populations?
3. Do people respond to relational value statements in a consistently different way than the New Ecological Paradigm (NEP) scale statements?

In the following sections, we outline our approach to data collection and analysis, present our results, and discuss the implications for environmental research and practice.

## 2. Methods

Our methods are comprised of two components: diverse sampling and comparing responses to different types of values. Our methods were reviewed and approved by University of British Columbia’s Behavioral Research Ethics Board (certificates H15-01325 and H14-02572). We obtained informed written consent from participants, all of whom were 18 or older, before they took the survey. We did not collect participant identifying information, nor was this part of the analysis. For our *sample*, we targeted three populations: farmers and international tourists in Costa Rica, and residents of U.S. coastal New England states. Our *surveys* included value/attitude statements followed by Likert scales to assess agreement/disagreement. Our *analysis* included factor analysis (for correlation in patterns of responses across questions and groups of questions) and calculating Cronbach’s alpha (for assessing consistency in responses across questions). Each step is described in more detail below.

### 2.1. Survey value statements and sample

We derived a list of value statements related to the environment including NEP, instrumental, relational, intrinsic, and values conveyed using metaphors.

The instrumental value statements were derived from concepts advanced in overviews of ecosystem services [15]. The NEP statements are a well-tested subset of the standardized NEP survey instrument [30]. The intrinsic, relational and metaphorically phrased value statements are derived from studies of cultural ecosystem services [19,23,33,34]. In retrospect, however, our measure of intrinsic value is limited in that we only used two items, both of which were negatively coded. Measuring intrinsic value in a more robust manner thus remains a challenge [8]. The metaphor statements are a rewording of four of the relational value statements. For example, the relational value statements express the relationship as a premise for a value statement, e.g., the kin metaphor statement, *kin_m*, is “I think about the forest/ocean and the plants and animals in it like a family of which I am very much a part.” Whereas the kin relational statement, *kin_r*, is “Plants and animals, as part of the interdependent web of life, are like 'kin' or family to me, so how we treat them matters”.

In all three surveys, the value statements (listed in Table 1) were placed in the final section of the survey, so as not to avoid priming responses in other areas of the survey protocol (involving research objectives not relevant here). Respondents were asked to evaluate the provided value prompts using a 5-point Likert scale (i.e., highly disagree = 1; highly agree = 5).

**Table 1:** Value statements used in surveys.

Variable	Category	Statement	Population	Reverse code
comm	Relational	There are landscapes that say something about who we are as a community, a people	F, T, MT	n
health	Relational	My health or the health of my family is related one way or another to the natural environment*	F, T, MT	n
iden	Relational	I have strong feelings about nature (including all plants, animals, the land, etc.) these views are part of who I am and how I live my life	F, T, MT	n
kin	Relational	Plants and animals, as part of the interdependent web of life, are like 'kin' or family to me, so how we treat them matters	F, T, MT	n
resp	Relational	How I manage the land, both for plants and animals and for future people, reflects my sense of responsibility to and so stewardship of the land	F, T	n
wild	Relational	I often think of some wild places whose fate I care about and strive to protect, even though I may never see them myself	F, T, MT	n
other	Relational	Humans have a responsibility to account for our own impacts to the environment because they can harm other people	F, T, MT	n
abuse	NEP	Humans are severely abusing the environment	F, T, MT	n
bal	NEP	The balance of nature is strong enough to cope with the impacts of modern industrial nations	F, T, MT	y
bau	NEP	If things continue on their present course, we will soon experience a major ecological catastrophe	F, T, MT	n
crisis	NEP	The so-called "ecological crisis" facing humankind has been greatly exaggerated	F, T, MT	y
spaceship	NEP	The earth is like a spaceship with very limited room and resources	F, T, MT	n
decade	Intrinsic	Humans have the right to use nature to meet our needs, even if this includes impacts that will take a decade or more to recover	MT	y
right	Intrinsic	Humans have the right to use nature any way we want	F, T	y
		*I think about the forest/ocean and the plants and animals in it like*: **		
iden_m	Metaphor	Something I identify with so strongly that it makes me, me	F, MT	n
kin_m	Metaphor	A family of which I am very much a part	F, MT	n
other_m	Metaphor	A world we must care for so that any damage doesn't also negatively affect humans who depend on it elsewhere	F, MT	n
resp_m	Metaphor	Beings to which we owe responsible citizenship and care	F, MT	n
extract	Instrumental (economic)	Natural resource extraction is necessary for countries to develop	F, T	y
clean	Instrumental (health)	It is important to protect nature so we have clean air and water	F, T	n
loss	Instrumental (use)	We can lose forests and wetlands, as long as we are keeping enough for the environment to function	F, T	y

* This statement was reversed for the M-Turk sample: “My health, the health of my family and the health of others who I care about is not necessarily dependent on the natural environment.” We do not recommend reversed coding this prompt because we later realized it caused confusion.

** The farmer sample responded to metaphorical statements related to forest. The M-Turk sample responded to metaphorical statements related to ocean. Tourists were not presented metaphorical statements.

F = Costa Rican Farmers, T = Tourists at San José airport; MT = Mechanical Turk respondents. Reverse codes were used when appropriate so high scores mean pro-environmental; y = yes; n = no.

Our aim with the populations sampled here is not to suggest they are representative, but to compare across different populations wherein other survey work was being conducted and thus some testing of these ideas could be done collaterally. Data collection included both online and paper-based surveys as appropriate for each group, specified below.

#### 2.1.1. Online survey

For the online sample, we used Amazon’s Mechanical Turk (M-Turk) system to enlist respondents, which has become a common recruitment method for experimental research [35,36]. Data outputs are generally just as reliable as those acquired with traditional recruitment methods [37]. We attempted to minimize selection bias in our sample by describing it on M-Turk’s HIT (Human Intelligence Tasks) list in general terms as a survey about preferences based on different text and image-based descriptions, without using any language related to ecosystems. The sample was limited to M-Turk workers who have mailing addresses in coastal New England states (Connecticut, Maine, Massachusetts, New Hampshire or Rhode Island). We targeted this geographic area because this survey also included questions assessing attitudes to a proposed renewable energy technology suited to this region—offshore wind farms. We collected self-reported demographic data from the sample to later compare it with census data to determine the extent to which this sample is representative of the population of these states. Upon survey completion, respondents were given a code to submit to the M-Turk system for payment. Respondents were paid $1 to take the 10–15 minute survey. Given that the typical M-Turk worker is willing to complete tasks for ~$1.40/hour [38], our payment was higher than the average reservation wage to expedite participant recruitment. Incomplete responses were discarded for a total of 400 M-Turk respondents.

#### 2.1.2. Paper-based survey

Two paper-based surveys incorporated value statements for two distinct populations in Guanacaste, Costa Rica. The first (*n* = 260) were international tourists in Costa Rica, who were randomly sampled in the Liberia Airport upon departure from the country. This airport primarily services the coastal tourist destinations and thus all international flights at this time were to the United States or Canada. All tourists in the departure lounge (i.e. those who arrived just in time to board did not have time to participate) during the week of May 25, 2015 were asked if they had travelled in the region, and if so if they were willing to participate in a survey. They were predominantly tourists from North America (and the U.S. in particular). The second group consisted of farmers in the Nicoya region (*n* = 253), mostly cattle ranchers, who spend a lot of time working the landscape, while also deriving their livelihoods directly from the environment.

We sought diversity across our three samples. Our expectation was that farmers would display a different profile with respect to their environmental values than the other two groups. We expected the international tourists to resemble the M-Turk population more closely, insofar as they include substantial representation of middle and upper income Americans. The farmers were randomly selected from lists provided by the agricultural extension agencies in the region, and the value statements for this group were included as part of a survey about environmental practices on the landscape more broadly.

### 2.2. Sampled population characteristics

Our M-Turk population was on average younger (32) than the tourist (45) or farmer populations (58)(Table 2). The tourists and M-Turk samples were a majority female while the farmers were mostly male (88% male) (Table 2).

**Table 2:** Demographic characteristics of our three samples.

Population	Socioeconomic Characteristics	Description	Percentage or Mean of Sample	Percentage or Mean from Reference Population
**M-Turk** **(*N* = 400)**	** **	** **	** **	**2014 US Census**
** **	**Income**	Annual household income before taxes	~$53,000	$66,200
** **	**Age**	Years old	32	40
** **	**Female**	Gender	0.59	0.51
** **	**Education**	Bachelor degree or higher	0.66	0.38
** **	**White**	Caucasian race	0.83	0.82
**Tourist** **(*N* = 260)**				
	**Income**	Income before taxes	~$75,000	
	**Age**	Years old	~45	
	**Female**	Gender	0.63	
**Farmer** **(*N* = 253)**	** **			
	**Education**	Bachelor degree or higher	0.15	
	**Age**	Years old	~58	
	**Female**	Gender	0.12	

### 2.3. Statistical analysis

We assessed the discrimination or uniqueness of each value category using factor analyses. Then we analyzed each using Cronbach’s alpha to test the internal consistency within value measures.

#### 2.3.1. Factor analysis

We calculated eigenvalues and created a scree plot to determine how many factors to include in our factor analysis. A common heuristic, which we used, is to retain components/factors with eigenvalues ≥ 1, which means that the component/factor accounts for as much or more variance as a single variable [39]. Our factor analysis investigated the structure of a set of variables to determine if there are clusters of correlation coefficients, which indicate latent variables, also called factors. This method derives a mathematical model from which underlying factors are estimated. Each latent variable is associated with some amount of the observed variable’s overall variance. Eigenvalues indicate the evenness in the distribution of the variances in the correlation matrix [39]. They measure the amount of the variance of the observed variables that a factor explains. If a factor has an eigenvalue ≥1, then it explains more variance than a single observed variable. In general, the factors explaining the least amount of variance are ignored.

**Fig 1:**
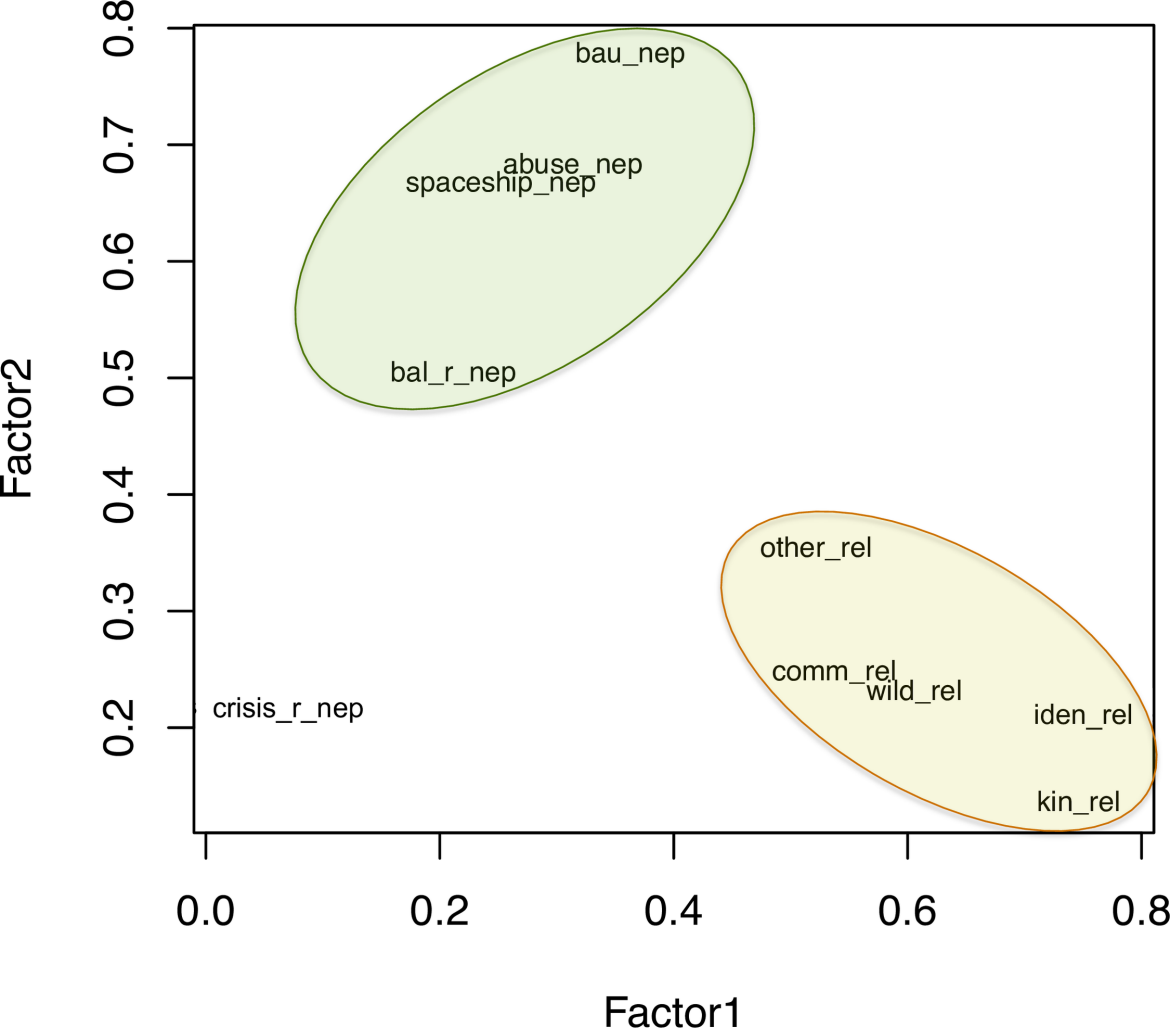
Graphical results of factor analysis.

In Factor Analysis, the amount of common variance is estimated by calculating communality values for each variable. This is usually done by calculating the squared multiple correlation of each variable with the others. We conducted an exploratory factor analysis with the hypothesis that responses to relational value statements comprise a factor distinct from responses to NEP statements (see Fig 1).

#### 2.3.2. Consistency measure: Cronbach’s alpha

We calculated Cronbach’s alpha for all of our social-ecological statements to determine the extent to which responses are consistent across NEP statements and relational statements. Cronbach’s [40] method is loosely understood as splitting a dataset in two in every possible way, then computing the correlation coefficient for each split. It is the most common metric of scale reliability [39].

## 3. Results

Our results suggest that relational value statements show internal coherence as a single dimensional construct, particularly when compared to responses to NEP prompts. We identified two factors and components when NEP and relational value statements were pooled and analyzed from our three populations using eigenvalues, a scree test, factor analysis. These two types of value statements showed high levels of internal consistency based on their high Cronbach’s alpha scores.

### 3.1. Two distinct factors based on eigenvalues and scree test

In order to understand distinctiveness in responses to types of environmental values and determine a reasonable number of factors to retain in our factor analysis, we calculated eigenvalues and conducted a scree test (S2 Fig and Table 3). Our scree plot, parallel analysis and optimal coordinates indicate that two factors ought to be retained for the factor analysis. The acceleration factor identifies where the slope of the curve changes most abruptly, which in our data, is directly after the first factor (see S2 Fig).

**Table 3:** Factor weights.

**Variable**	**Factor 1**	**Factor 2**
	**Relational**	**NEP**
comm_rel	0.54	
wild_rel	0.61	
iden_rel	0.78	
kin_rel	0.75	
other_rel	0.52	0.35
abuse_nep	0.31	0.68
bal_r_nep		0.5
spaceship_nep		0.67
bau_nep	0.36	0.78
crisis_r_nep		
		
	**Factor 1**	**Factor 2**
	**Relational**	**NEP**
**Eigenvalues/SS loadings**	2.43	2.11
**Proportion Variation**	0.24	0.21
**Cumulative Variation**	0.24	0.45

### 3.2. Factor analysis results: NEP is distinct from relational value

**Fig 2:**
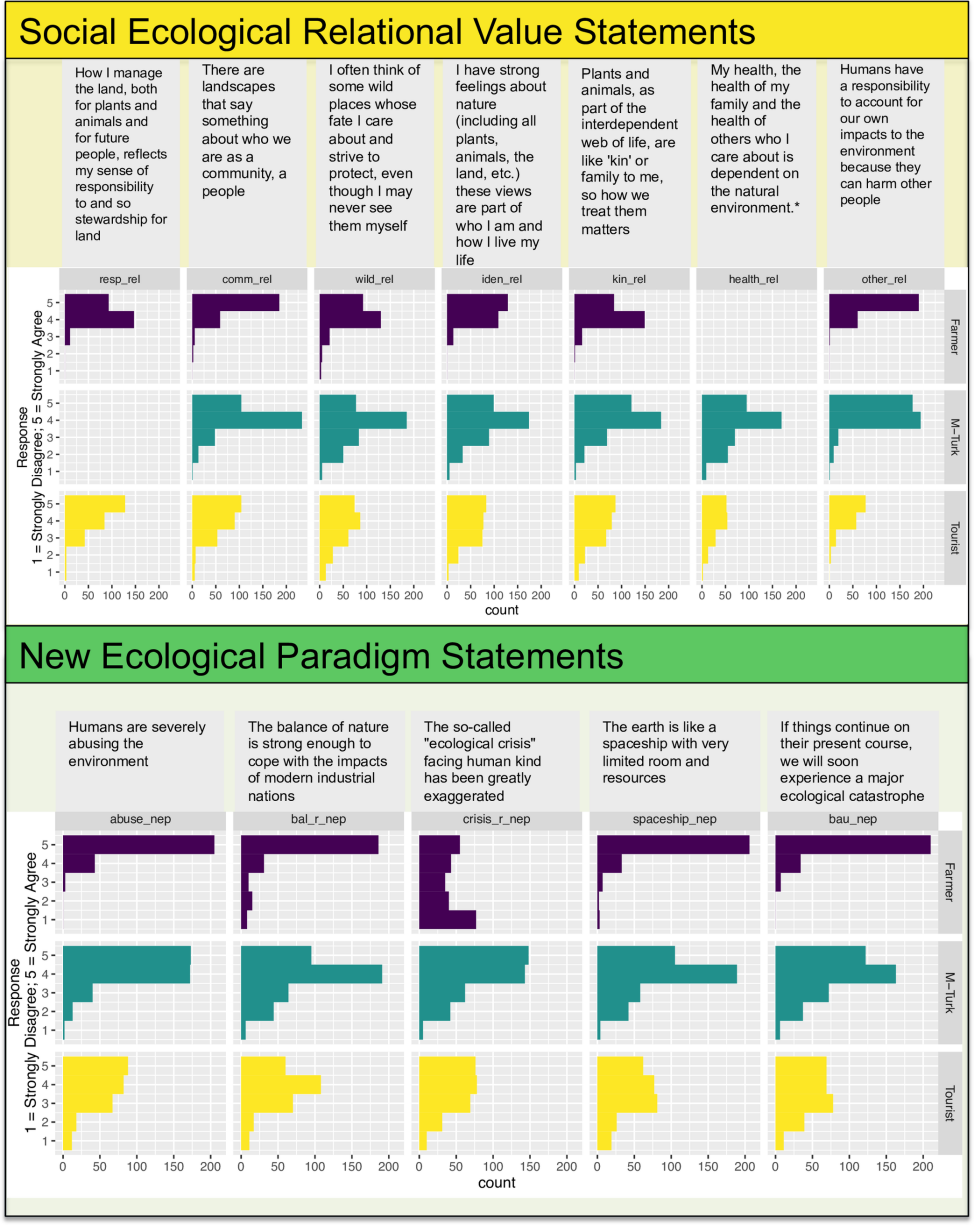
Mean and distribution of responses to relational value prompts and New Ecological Paradigm Statements. The sample includes Costa Rican farmers (n = 253), tourists in Costa Rica (n = 260) and US M-Turk workers (n = 400). *The health_rel prompt for the M-Turk population was worded “My health, the health of my family and the health of others who I care about is not necessarily dependent on the natural environment.” Scores were reversed for this population when included in the analysis.

Our factor analysis shows that survey takers responded differently to relational value prompts than NEP statements (Table 3 and Fig 2). The proportion of variation attributed to Factor 1, the “Relational” Factor (0.24), is higher than the proportion attributed to Factor 2, “NEP” factor (0.21).

Our factor analysis results show a grouping of the relational questions that is distinct from the NEP statements. The crisis NEP statement is an outlier in the pooled data (Fig 1), which is discussed in greater detail in the discussion.

### 3.3. High levels of agreement and consistency with types of environmental value statements

Strong relational value statements resonate with diverse populations based on how the average response to relational value and NEP statements was 4 (Agree). The responses to NEP statements, on average, reflect relatively high ecological concern (see Table 4). NEP responses were consistent (Tourist *α* = 0.79 and M-Turk *α* = 0.84), except for Costa Rican farmers (*α* = 0.35), largely due to the farmers’ wide variation in response to the “crisis” prompt (*The so-called "ecological crisis" facing humankind has been greatly exaggerated*, see Table 1). We did not include instrumental or intrinsic value statements when calculating *α* because of the limited number of statements in these categories (except for M-Turk population as reported in S3 Fig).

**Table 4:** Cronbach’s alpha, mean response and standard deviation of responses across value statements.

	Cronbach’s alpha	Mean	Standard deviation
**NEP (5)**			
Full dataset	0.73	4.0	0.75
Farmers	0.35	4.3	0.49
Tourists	0.79	3.7	0.81
M-Turk	0.84	4.0	0.74
**Relational (6)**			
Full dataset	0.80	4.0	0.68
Farmers	0.73	4.4	0.43
Tourists	0.79	3.9	0.75
M-Turk	0.79	3.9	0.61

**Fig 3:**
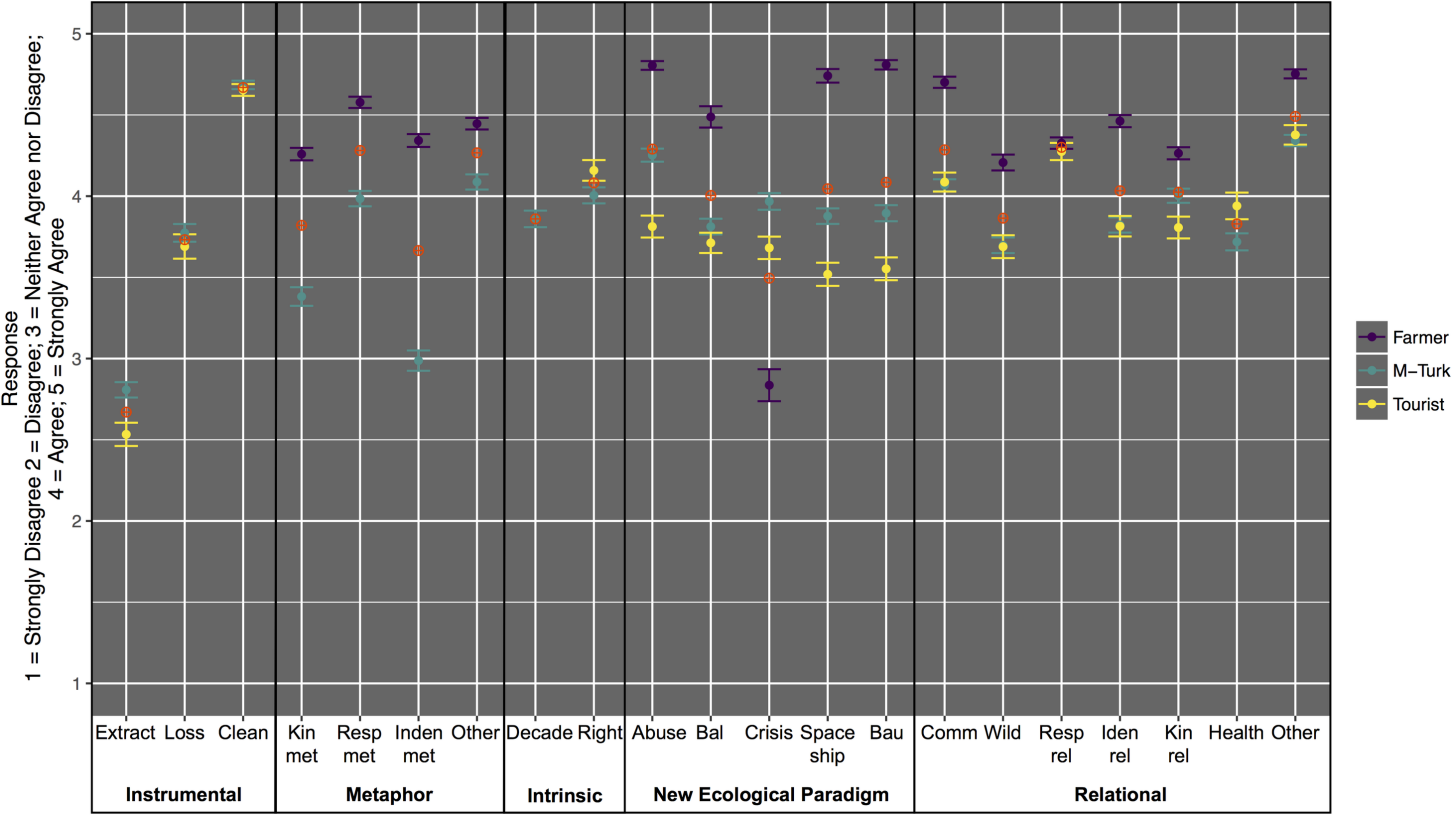
Mean response with standard errors to value prompts across three populations. Red circles indicate the mean response across the populations for each value statement.

Costa Rican Farmers responded differently to our value statements than the M-Turk and Tourist samples. The Farmers on average responded with higher levels of agreement to relational value prompts (mean = 4.4) as compared to Tourists (mean = 3.9) and M-Turk workers (mean = 3.9) (Table 4). Farmers on average scored higher on the NEP scale (mean = 4.33) than Tourists (mean = 3.65) and M-Turk workers (mean = 3.96) (Table 4, Figs 2 and 3). The relational and NEP statements as well as the distribution of Likert-scale responses across the three populations is shown in the histograms in Fig 3. The x-axis is the number of respondents and the y-axis is the items of the Likert scale (1 means strongly disagree to 5 meaning strongly agree).

A shown in Fig 3, the M-Turk and tourist populations responded similarly to the instrumental value statements (the standard errors overlap for 2 out of 3 instrumental value prompts). Costa Rican farmers agreed more strongly with the metaphorical statements than the M-Turk population. Except for the “crisis” statement, Costa Rican farmers scored the highest on the NEP scale, followed by M-Turk then the Tourist population. The M-Turk and Tourist populations responded similarly to the relational value prompts and lower than the farmers (except for the similar responses to the responsibility prompt, “resp_rel”).

Out of all of the environmental value statements that we tested, the highest average response for the M-Turk and Tourist population was agreement with an instrumental value: It is important to protect nature so we have clean air and water (“Clean”). Two NEP statements (“BAU” and Abuse”) ranked highest for the farmer population as shown in Fig 3 and Table 5. See S4 Fig for the distribution of responses across the three populations and value statements.

**Table 5:** Top six mean responses to environmental value statements across three populations. The top four farmer scores are not statistically different from each other, effectively all being tied for first, comm_rel is statistically different from the first two, bau_nep and abuse_nep.

Rank	M-Turk	Tourist	Farmer			
1	Clean (4.69)	Clean (4.6)	BAU (4.81)			Instrumental
2	Other (4.34)	Other (4.4)	Abuse (4.81)			Intrinsic
3	Abuse (4.25)	Responsibility (4.3)	Other (4.75)			Metaphor
4	Other (4.09)	Right (4.1)	Spaceship (4.74)			NEP
5	Community (4.07)	Community (4.1)	Community (4.70)			Relational
6	Right (4.00)	Health (3.9)	Responsibility (4.58)			

## 4. Discussion

This research is a first step in seeking to operationalize the “relational values” construct in a survey form in reference to other widely used constructs (intrinsic and instrumental) and a measure of environmental beliefs already widely used (NEP). The following sections discuss the research questions in turn. The first is associated with the relational concept in general, namely that diverse populations agree with the statements, suggesting that what we refer to as a “relational framing” (in terms of the phrasing rather than as an experimental design) is widely resonant. The following two sections discuss how responses differed between the relational statements and the NEP, followed by how there was consistency in responses to the relational statements, which could lead to treating this set of statements as an index. Also, the theoretical and policy implications of these findings and proposed paths forward are discussed.

### 4.1. Diverse populations tend to agree with strong relational value statements

Agreement with relational values was higher than anticipated across populations. The mean response for all three of the populations to the relational value statements was 4 (see Table 4, Figs 2 and 3) which is equivalent to “agree” on the Likert scale. The average for each relational value prompt was higher than 3.6. We expected somewhat lower means given the explicit nature of the social-ecological linkage and our deliberate attempt to phrase the prompts strongly to foster variation in our sample. The relational prompts push the bounds of how people think about the environment in relation to themselves–such as thinking of wildlife as kin and considering the environment as part of their identity. Although environmentalism may have become marginalized in the last decade [41], these social-ecological relational statements clearly resonate with our M-Turk, tourist and farmer samples (i.e., respondents tend to agree and strongly agree with the value statements) (Fig 2).

The comparison between the relational value and metaphor statements is instructive, suggesting that although social-ecological *relations* are lower in North American populations, associated *values* remain strong in the populations we surveyed. M-Turk samples tend to be comprised of ~90% urban residents [42]. The farmers’ responses to the metaphor statements were significantly higher than the M-Turk responses, and in the same range as their relational responses. The M-Turk population responses to the metaphorical statements were significantly lower than both the farmers and the M-Turk relational responses (Fig 3). We speculate that the farmers are comfortable talking about nature in a deeply relational way, while the M-Turk population is likely less comfortable with such ‘relationality’, *but can still agree* with the moral conclusion expressed in the relational statements. We view this as further indication that a relational framing may be an accessible way to engage diverse parties for the purpose of conservation, including those who do not have an ecocentric worldview.

Relational value responses do not have the highest average among the types of value statements in the three populations (Table 5). Out of the 17 statements presented to all three populations, the overall highest ranked statements (in two of the three populations: tourist and M-Turk) was the “clean” statement: “It is important to protect nature so we can have clean air and water.” We classified “clean” as an instrumental statement (Table 1), but it is not narrowly self-oriented since it implicitly includes concern for the well-being of others. The highest overall statement for the farmers was “bau” (“If things continue on their present course, we will soon experience a major ecological catastrophe,” i.e., business as usual). However, since the farmer responses were so high overall—their top 5 responses averaged over 4.7, meaning that the majority of respondents answered 5—the differences between the top 5 are not significant (with the exception of the fifth being different from the first and second rank based on t-test results—Table 5), thus the top four could all be considered a top response.

It is not surprising that relational values were not noticeably higher in the farmer population as compared to their NEP scores. We perceive the benefit of relational values is that it may allow people to express environmental concern that they otherwise would not (on a scale like the NEP, for example). For people with already high environmental values, it is not surprising they score equally high in this alternative framing.

The top six overall mean scores of our three populations are depicted in Fig 3. For the tourist population, four of the top six mean scores were relational statements. All three populations included the “community” statement as the fifth highest. The M-Turk and farmer population shared two of the top five (“community” and “other”). The community statement refers to recognizing the uniqueness associated with place, where as “other” refers to responsibility to reduce environmental harms felt by people elsewhere. All six relational statements are represented in the top 6 value statements when all three populations are combined, suggesting 1) there is resonance of relational statements in general, and 2) different aspects of relational values resonate with different populations, that is, averaging across different populations we see high levels of agreement with several relational statements.

### 4.2. Relational value responses are distinct from NEP

The factor analysis (FA) (Table 3, Fig 1) reveals a distinction between relational value responses and the NEP. Additionally, this analysis allows comparison across statements and sets of question to determine the consistency with which individuals and subpopulations responded to the survey, enabling underlying factors to emerge (Child, 1970). The statements cluster in the factor analysis differently as individual populations (S1 Fig) as compared to pooled results (Fig 1) but in all four cases the distinction between the two sets is clear. Examining uniqueness of the relational statements as compared to the NEP, the former has a higher proportional variation in the pooled data set (Fig 1), meaning the relational statements are more tightly knit as a group than the NEP. The relational statements fall into distinct components or factors, which supports the hypothesis that the relational framings induce a different but coherent response pattern. This response is also consistent, as evidenced by the high α across the relational statements (Table 4).

### 4.3. Relational statements can be a single construct and have potential as new index

Our Cronbach’s alpha scores suggest, that the six relational values statements cluster together strongly as an index. Each statement captures a different aspect of relationships with nature, and are not intended as multiple expressions of the same idea, so it is interesting how strongly the statements do cluster. This cluster result was echoed in the NEP for tourists and the M-Turk population, with α scores of 0.79 and 0.84 respectively, whereas the farmers had a score of 0.35. The exception driving this unexpected result is the farmer response to the crisis statement; the widely distributed spread of responses for this statement can be seen in Fig 2. We expected that respondents with a tendency toward an ecocentric worldview to score low for this “crisis” statement, and those with anthropocentric worldview to score high. The farmer results across all statements (see Figs 2 and 3) demonstrate consistently high mean responses that are also statistically higher than the other two populations as noted by the t-test results. This rural population of predominantly small-holder Costa Rican farmers are reliant upon environmental conditions for their livelihoods, and thus their strong environmental values (as understood through all of their responses) are expected. This is reflected in their high scores, and in the case of the “abuse” statement, statements where not a single farmer answered lower than a 4 (i.e. all respondents answered agree or strongly agree). This brings in the question of why the farmers did not follow the pattern of eco-centrism in their NEP results, which is associated with strong environmental values and evident here.

The anomaly is due to the response to the crisis statement. We propose two possible explanations: wording and lack of urgency. The statement reads, “the environmental crisis is greatly exaggerated,” with the expectation that those answering 4 or 5 (agree or strongly agree) are not as concerned about the environment as 1 or 2 (strongly disagree or disagree). It is conceivable in this region that those answering with a 4 or 5 are deeply concerned about environmental issues, but it is such a focal point that from their perspective it is overemphasized. That is, their agreement with the statement speaks to the strong wording of “great exaggeration” rather than suggesting environmental issues are not present. An additional possibility is that these farmers are better equipped to cope with change than their neighbours, thus reducing an overall sense of urgency. All farmers who responded 4 or 5 to this question (about 30%) responded in the expected NEP pattern matching an ecocentric worldview on the other NEP prompts, so we do not believe that these farmers lack ecocentric views. In any case, this result did not impact the analysis dramatically insofar as the responses to NEP were distinct from response to relational prompts across the populations (see Table 3 and Fig 1).

Farmer anomaly aside, the inclusion of NEP statements enabled us to demonstrate that, for the most part, the statements correlated as expected, and our populations behaved consistently with NEP experiments elsewhere. The high Cronbach’s alpha scores across the individual populations and all three pooled indicate that people responded consistently to the NEP and relational statements. In general, an alpha of 0.7 and higher is considered strong [43]. Our high relational value alpha of 0.8 suggests there may be potential to generate a scale or index of the statement set, and we consider the development of such an index an avenue for future research.

### 4.4. Theory implications

As proposed in the introduction, we see potential to utilize relational values as a means to solidify or enhance connections to the natural world, by invoking other held values that are not necessarily environmental. That is, instead of thinking of nature as external or “outside of oneself,” by connection to family, places we care about, and human well-being, ‘nature’ becomes part of an individual’s realm of care.

We refer to relational values as a framing rather than as a novel way of thinking about the environment to recognize and emphasize this is not new conceptual territory. Environmental values have been studied extensively, along with their connections to attitudes and behaviours (Stern et al., 1995, Dietz et al., 2005, Spash et al., 2009). Likewise, the attributes captured by our value statements were selected based on existing studies and theory that suggest associations with family, community, and identity are powerful and meaningful ideas that people will take action to protect and uphold [44,45]. Our eventual aim is to examine whether this new value-frame can augment and support existing theories of value that posit pathways between different categories of values, beliefs (in the NEP sense of the word) and behaviour. This study is not sufficient to do so, but our data points to encouraging possibilities along this path. Here we discuss how we envision the relational framing to contribute to the values, beliefs and norms framework [46,47].

**Fig 4:**
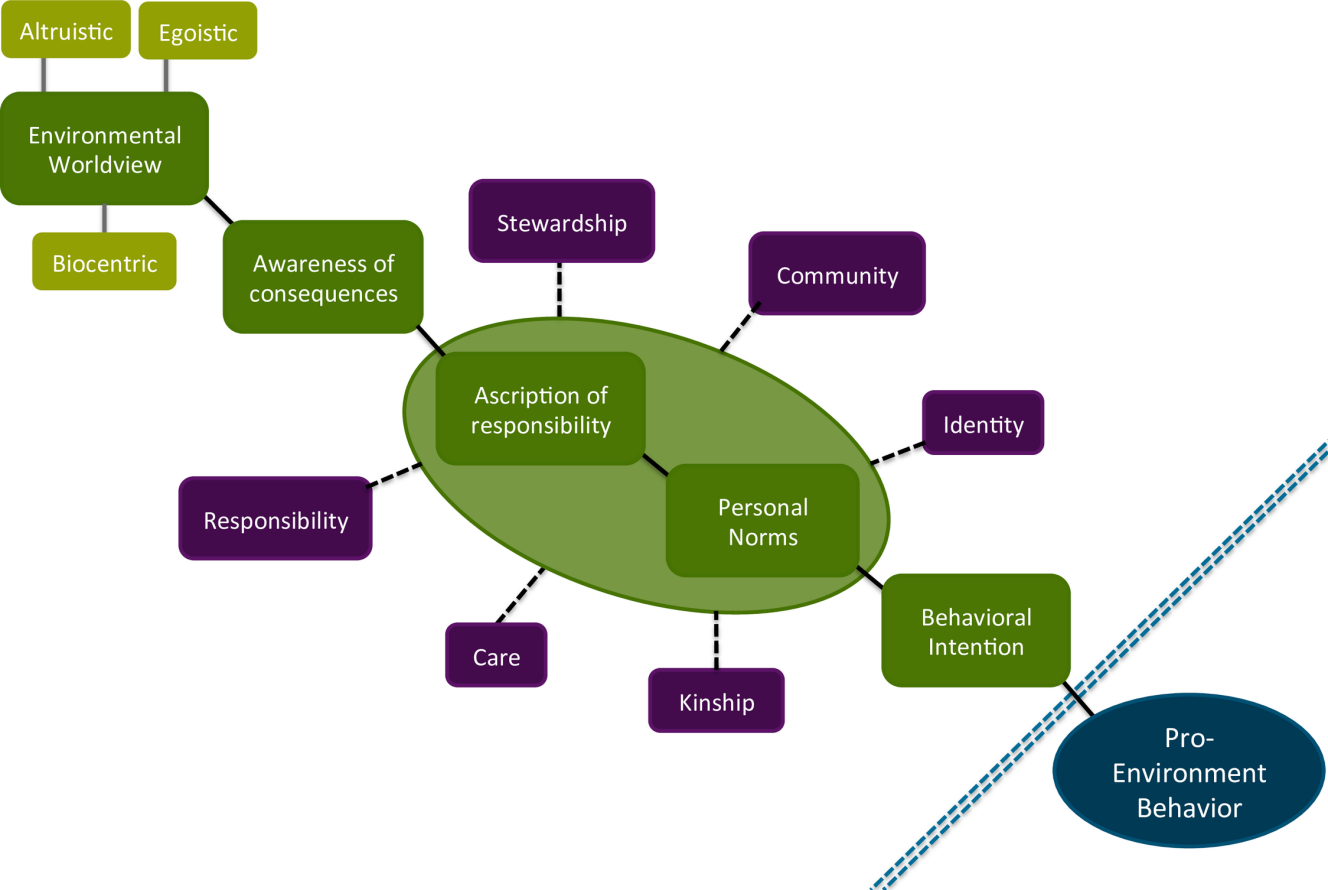
Value-belief norm model (green) with our proposed ways in which relational framings (purple) could influence steps of this pathway (black dashes). We acknowledge the variety of barriers between behavioral intention and pro-environment behavior (dashed blue line).

Values, beliefs and norms (VBN) theory suggests that there are relationships linking 1) the acceptance of basic values; 2) believing that something important is threatened; and 3) the activation of a personal norm (obligation) to take action to restore those values [46,47]. VBN posits that values influence our worldviews, which in turn influence our beliefs of how environmental change has consequences for our values, and these beliefs underlie norms from which we take action [47]. Fig 4 outlines the VBN theory in green, and highlights in purple how we imagine our selected relational value dimensions contribute to this pathway. Our results are far too limited and preliminary to support the hypothesis that social-ecological relational framing influences behavioural intention (let alone behavior—even the VBN theory does not claim to explain pro-environmental behaviour), but we propose future studies to test this.

Fig 4 highlights where our relational value framings might support the theorized linkages in the VBN. We propose that by leveraging some components of the model—namely responsibility to others (both human and non-human) and personal norms—the pathway may be strengthened or other components may be bypassed. For example, a mother with anthropocentric views and little understanding of consequences of a particular threat where she lives (such as climate change influencing flooding), may still be induced to support a new coastal protected area in her community, if doing so is consistent with notions of good parenthood or citizenship.

Reflecting upon our results in the context of this diagram, we note that the highest scores from the relational statements were those that referred to groups in which they are a part or to which they feel a sense of responsibility, including family and community. Psychological evidence points to the importance of in-groups, social norms, and peer-pressure to influence behaviour, both in general and with pro-environmental behaviours specifically [48,49]. While instrumental and intrinsic values tend to focus on individual ways of thinking about the world, we propose relational framings have the capacity to establish or enhance social influences that encourage action.

### 4.5. Policy and practical implications

Governments, NGOs, and decision-making bodies wrestle with how to effectively engage communities in environmental decision-making processes [50]. Regulatory bodies and environmental impact assessment require consultation, yet assessments tend to focus on biophysical impacts and have struggled to capture cultural ecosystem services, due to their less tangible and less quantifiable nature. We propose there is a gap in the existing tools that explore and explain values on how we relate to the environment. Relational values may be used to frame or facilitate discussions in decision-making processes connecting environmental change to tangible and intangible values. Here again we refer to framing in terms of a value construct, rather than comparative framing used in experimental designs. Methods to assess social-ecological relational values could be further refined to characterize how communities or individuals think about the environment. Invoking relational values may be key to reframing conservation policy approaches [51].

Framing conservation with relational values may offer more powerful leverage for conservation than emphasis on instrumental or intrinsic values. Intrinsic values alone are enough to motivate only a minority of people to achieve conservation goals [52]. A broader array of people can be motivated by appeals to financial benefit and self-interest in the name of conservation, but such appeals reinforce ‘extrinsic’ values—those associated with the pursuit of prestige, power, image and status. Psychological research has shown that reinforcement of extrinsic values can suppress intrinsic values, which are linked to concern for others and the environment, kindness, understanding, appreciation, tolerance and protection of people and nature [10,48,53–55]. Furthermore, an instrumental-value basis for conservation tends to only motivate conservation that is demonstrably useful [56,57].

Relational value statements could be a part of how the International Union for Conservation of Nature’s Key Biodiversity Areas partnership conducts biodiversity documentation, which would include consistently collected information that assists policy advocacy on-site, as well as broader analysis to prioritize areas for conservation. This partnership, as just one example of a potential application of relational values, identifies important sites for various taxa, and is currently consolidating a variety of partners to create a framework for assessment (threats, associated ecosystem services, etc.) [58]. These data could support prioritizing conservation actions and policies that resonate with people locally. In a similar vein, diverse conceptualizations of values are incorporated in the conceptual framework of the International Panel for Biodiversity and Ecosystem Services (IPBES). Relational value statements may help operationalize these diverse conceptualizations in the planned regional assessments.

We anticipate the concern that employing community values or framing options could be used to merely leverage instrumental values. Though we do not explicitly test that, our hypothesis relates to encouraging environmental values by anchoring them to something they already care about and with which they already identify (e.g., community, family). Our intention is not to find another avenue to “sell” the environment and its associated benefits to a broader audience. As highlighted by Chan et al., “To be more than mere marketing, environmental management must reflect on and possibly rethink conservation in the context of local narratives and struggles over a good life” [9](p. 1464).

### 4.6. Proposed paths forward

Our assessment of social-ecological and in particular relational values resulted in a preliminary scale that can help launch future research. Our objective was not to create a new, universally valid scale for social-ecological relations. Although we capture diverse types of relational values, we do not claim to have captured all aspects of “relationality.” We acknowledge there may be different and/or additional statements that could enrich a social-ecological relational index. We can imagine several research trajectories, stated below, as well as how other future research may augment the ambitions of this preliminary study.

- **Expand and refine social-ecological relational statements**. Our six relational statements are not comprehensive. We can imagine further dimensions to be tested, such as the extent to which natural elements contribute to a sense of belonging. Index development in the psychological literature entails including more overlap between statements to probe similar themes in multiple ways and test agreement with various statements in different cultural settings (if universality–to the degree it is possible–is the goal). The list should be refined until there is greater certainty of its appropriateness and accuracy for assessing the presence and strength of relational values.
- **Explore social-ecological relational values with other methods.** Surveys can be useful, but other methods, such as interviews and focus groups, can help delve into the complexity and context-specific dimensions of social-ecological relational values.
- **Use social-ecological relational value statements as an index in before/after or control/impact studies**. Such research would shed light on values in the context of various environmental management and conservation interventions.
- **Embed social-ecological relational values research in scenarios with real-world constraints.** We envision empirical testing of relational values in the context of tradeoffs and/or external constraints, including scenarios or choices to more accurately reflect the types of decisions people make on a daily basis. One particular set of people whose behaviours are of particular interest includes consumer responses to relational framings, and testing consumption behavior when the disconnect between consumption practices and environmental impact are made more explicit.

## 5. Conclusion

The study provides preliminary empirical evidence of widespread support for social-ecological relational values, an emergent topic in conservation [9,51]. We foresee diverse paths forward to test relational values as a means of overcoming the instrumental vs. intrinsic value of nature debate.

Self-interest tends to prevail when instrumental values dominate communications, campaigns and debates [10]. Instrumental values, however, are one type of the various values that can come into play when we make decisions. Insights from cognitive psychology highlight how we often make decisions and act based on affective responses to situations rather than mental calculations of utility associated with different outcomes [11,12]. Similarly, while we acknowledge the logic behind instrumental justifications for biodiversity conservation, studies show numerous other values, beliefs and attitudes motivate conservation action, including, but not limited to, identity and social norms, biophilia, altruism and notions of reciprocity. Leveraging these motivators in relational terms might engage more people and enable individuals and communities to rethink conservation in the context of local narratives and what it means to pursue a good life, which goes far beyond focusing on instrumental values [9].

This study suggests a relational value framing as a new direction for innovation when it comes to ecosystem service assessments and designing conservation initiatives. This could not only inform, but also inspire the action necessary to cultivate a future better for humans and other species.

## Supporting information

S1 Fig Factor analysis by population with results from tourist, farmer and M-Turk sample. (PDF)

S2 Fig Scree plot. Scree plot including responses to five NEP statements and six relational value statements across all three populations. Parallel analysis, optimal coordinates and acceleration factors are different methods to determine the number of factors to retain.
(PDF)

S3 Fig M-Turk Cronbach’s alphas. (PDF)

S4 Fig Distribution of responses to value prompts. (PDF)
