# Supplementary material for: Relational values resonate broadly and differently than intrinsic or instrumental values, or the New Ecological Paradigm
Source: PLoS One. 2017 Aug 30;12(8):e0183962. doi: 10.1371/journal.pone.0183962 (PMC5576695; doi:10.1371/journal.pone.0183962)
Supplement: S1 Fig — (PDF) [file pone.0183962.s001.pdf]

S1 Fig Factor analysis by population

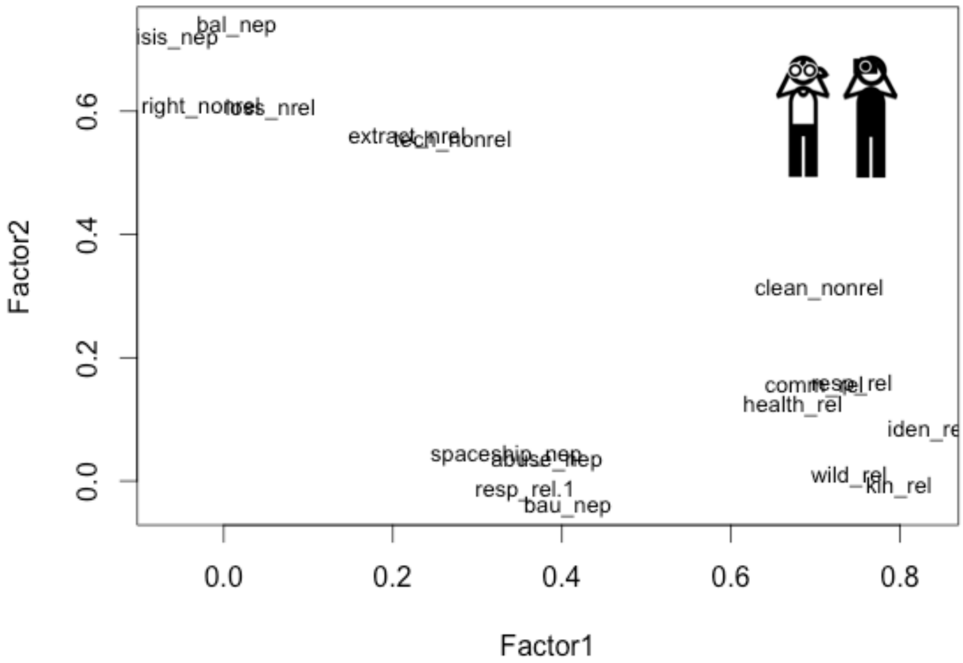

Factor analysis results from tourist sample

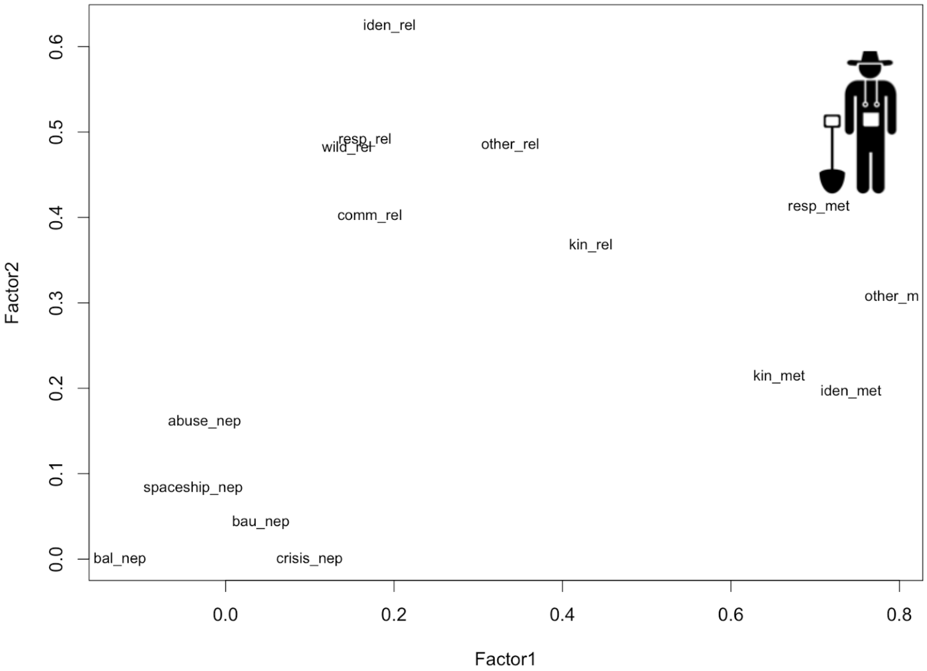

Factor analysis results from farmer sample

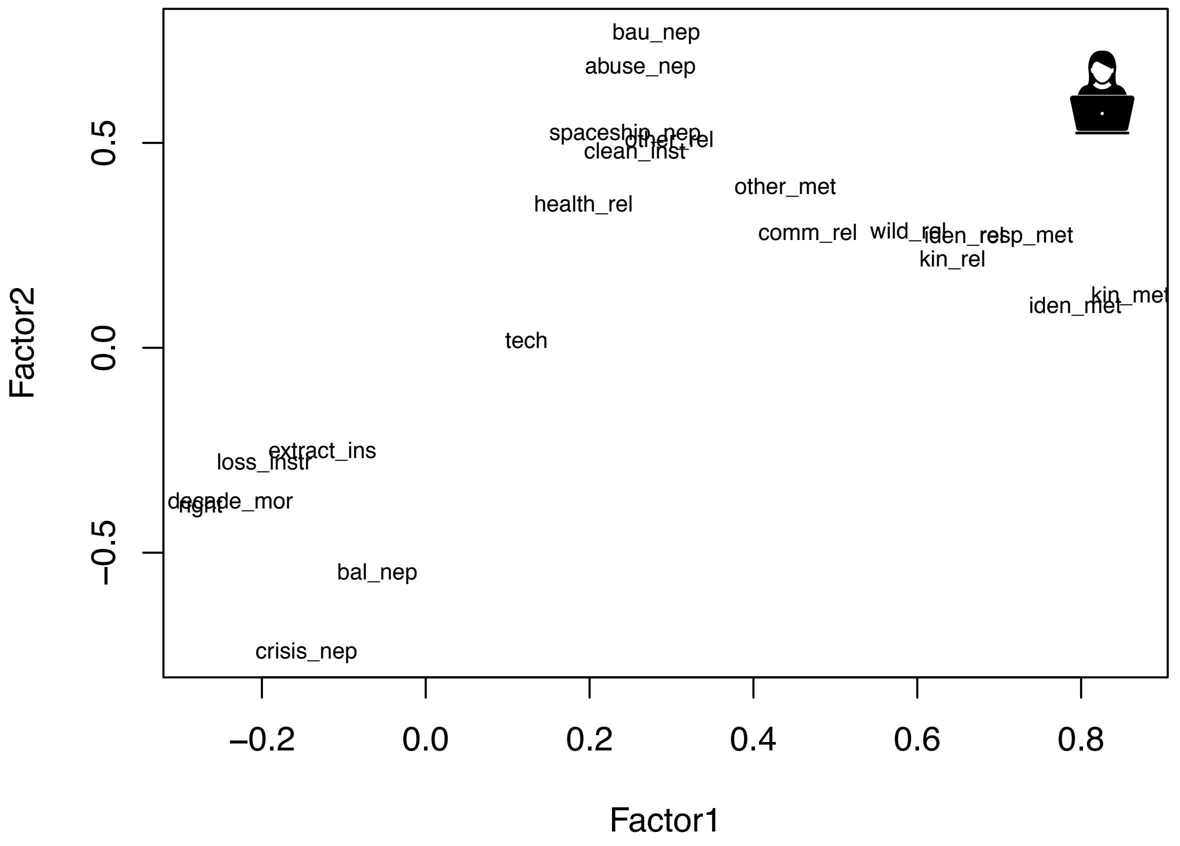

Factor analysis results from M-Turk sample
