# Supplementary material for: Relational values resonate broadly and differently than intrinsic or instrumental values, or the New Ecological Paradigm
Source: PLoS One. 2017 Aug 30;12(8):e0183962. doi: 10.1371/journal.pone.0183962 (PMC5576695; doi:10.1371/journal.pone.0183962)
Supplement: S2 Fig — Scree plot including responses to five NEP statements and six relational value statements across all three populations. Parallel analysis, optimal coordinates and acceleration factors are different methods to determine the number of factors to retain. (PDF) [file pone.0183962.s002.pdf]

S2 Fig Scree plot

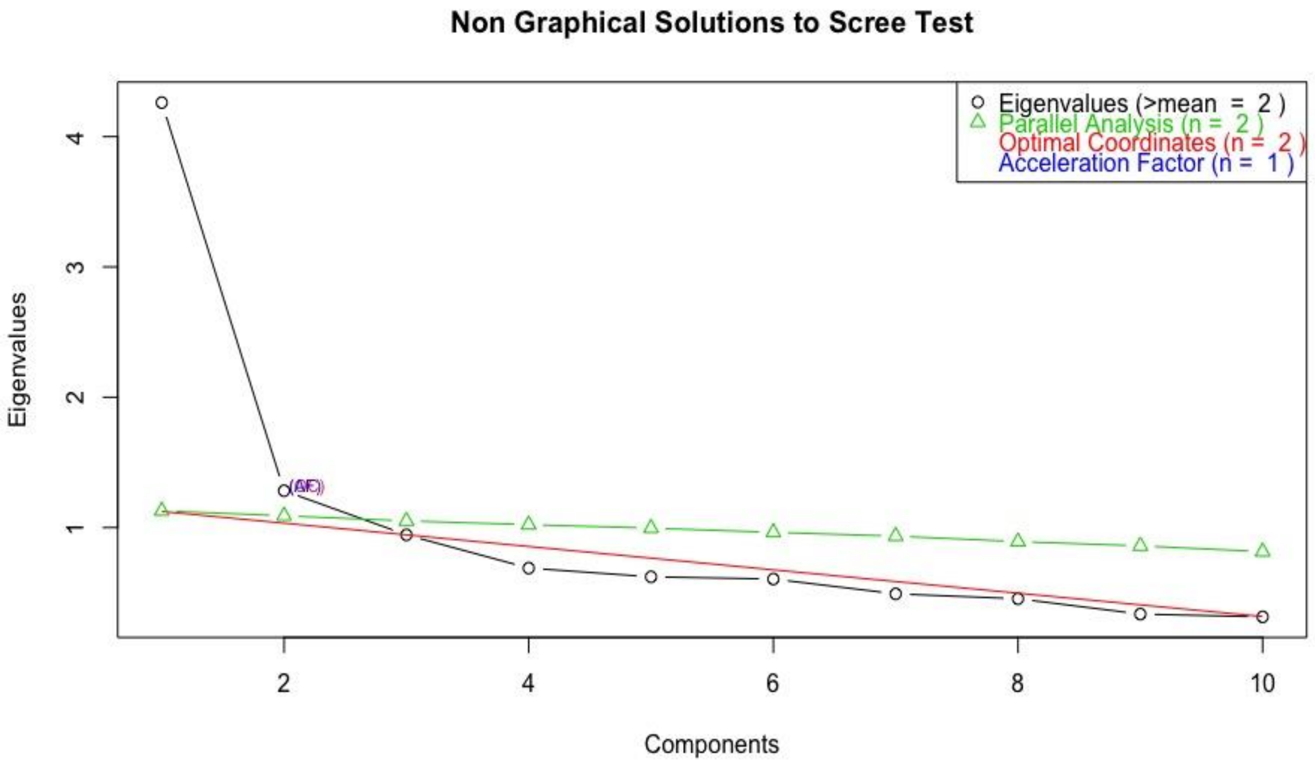

Scree plot including responses to five NEP statements and six relational value statements across all three populations. Parallel analysis, optimal coordinates and acceleration factors are different methods to determine the number of factors to retain.
