# Supplementary material for: Relational values resonate broadly and differently than intrinsic or instrumental values, or the New Ecological Paradigm
Source: PLoS One. 2017 Aug 30;12(8):e0183962. doi: 10.1371/journal.pone.0183962 (PMC5576695; doi:10.1371/journal.pone.0183962)
Supplement: S3 Fig — (PDF) [file pone.0183962.s003.pdf]

S3 Fig M-Turk Cronbach's alphas

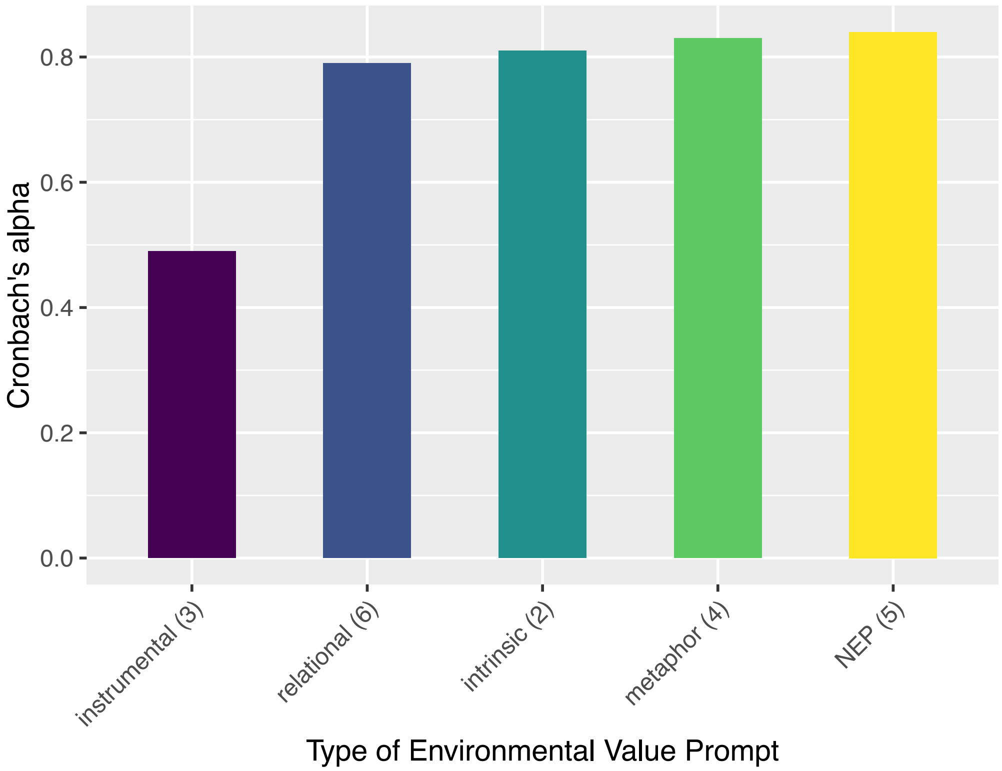

Cronbach alphas for M-Turk population. Note the different number of prompts in each category as shown in parentheses after each environmental value type. We suggest testing additional intrinsic and instrumental value prompts.
