# Supplementary material for: Relational values resonate broadly and differently than intrinsic or instrumental values, or the New Ecological Paradigm
Source: PLoS One. 2017 Aug 30;12(8):e0183962. doi: 10.1371/journal.pone.0183962 (PMC5576695; doi:10.1371/journal.pone.0183962)
Supplement: S4 Fig — (PDF) [file pone.0183962.s004.pdf]

S4 Fig Distribution of responses to value prompts

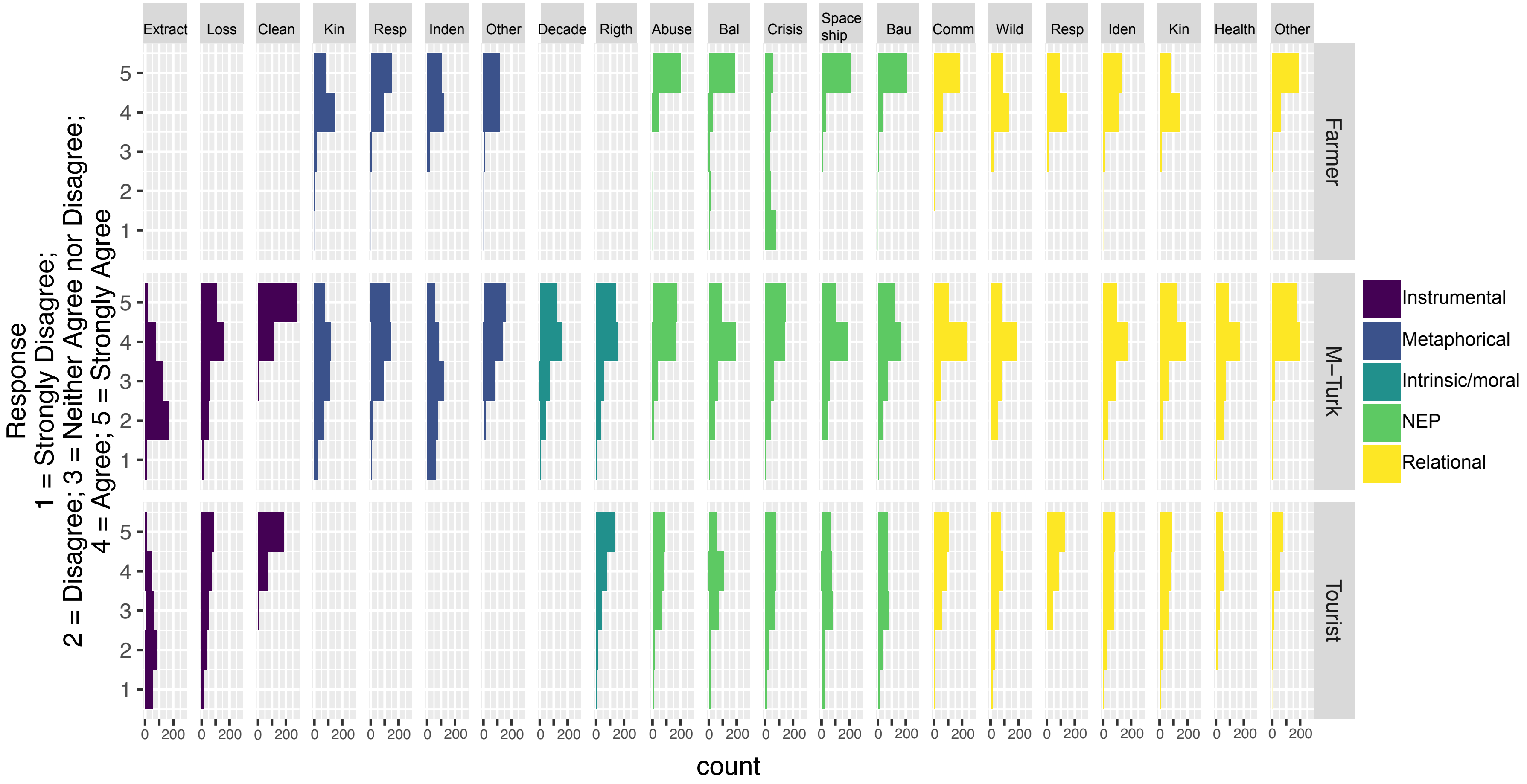

Three populations (Costa Rican Farmers, M-Turk workers and tourists in Costa Rica) responded to five types of environmental value statements in surveys.
